# A case report of the successful administration of clozapine in the face of myocardial infarction, pulmonary embolism and hyperlipidaemia resulting in the termination of long-term seclusion

**DOI:** 10.1186/s12888-018-2001-7

**Published:** 2019-01-23

**Authors:** Alex Till, Ed Silva

**Affiliations:** 1 School of Psychiatry, Health Education North West (Mersey), Liverpool, L3 4BL UK; 2 Ashworth Hospital, Mersey Care NHS Trust, Parkbourn, Maghull, Liverpool, Merseyside L31 1HW UK

**Keywords:** Clozapine, Nasogastric clozapine, Schizophrenia, Psychosis, Myocardial infarction, Pulmonary embolism, Hyperlipidaemia

## Abstract

**Background:**

Cardiometabolic health significantly impacts on the mortality of people with severe mental illness. Clozapine has the greatest efficacy for Treatment Resistant Schizophrenia (TRS) but the greatest negative impact on cardiometabolic health. Balancing the risks and benefits of treatment, dignity, autonomy, liberty, mental and physical health can be challenging, particularly when imposing interventions with potentially life threatening adverse events, such as clozapine. We describe the successful administration of clozapine in the face of myocardial infarction, pulmonary embolism and hyperlipidaemia resulting in the termination of long-term seclusion for a gentleman with TRS in high secure psychiatric services.

**Case presentation:**

The impact of clozapine on a 44-year-old gentleman with TRS, extreme violence requiring physical restraint and long-term segregation, and numerous other significant physical health complications is described. He had metabolic syndrome; a poor diet, sedentary lifestyle, Body Mass Index (BMI) of 31.5, poorly controlled lipids and had smoked heavily since childhood. During preparations to initiate clozapine, he suffered a myocardial infarction and pulmonary embolism. His compliance with secondary prevention medications was poor due to paranoid persecutory and somatic delusions. Despite these concerns, nasogastric administration of clozapine was approved and prescribed within nine months of his myocardial infarction and a month from his pulmonary embolism but was ultimately not required. Accepting oral medication, his mental state made a rapid and dramatic improvement. After spending 1046 days in seclusion, this was terminated 94 days after clozapine initiation. He has been compliant with all medications for 24 months, had no incidents of violence or seclusion, and has been transferred to medium secure services. His physical health stabilised despite continuing to lead a sedentary lifestyle and remaining obese (BMI of 35). He developed hypertension, Type II Diabetes Mellitus and his triglycerides rose to 22.2 mmol/L in the same month after clozapine initiation. However, with pharmacological intervention, 24 months later these are controlled, and he has had no further thromboembolic events.

**Conclusions:**

We highlight that despite significant physical health concerns, clozapine can be successfully initiated and safely prescribed with a significantly positive effect on both the psychiatric and holistic care of patients with treatment resistant schizophrenia.

## Background

Clozapine is licensed in patients unresponsive to, or intolerant of, conventional antipsychotic drugs. In Treatment Resistant Schizophrenia (TRS), defined as an inadequate response to at least two antipsychotic drugs at the maximally tolerated dose within the recommended therapeutic range in trials lasting six weeks or more, clozapine is the only drug treatment likely to be effective [1]. For these patients, 30% would be expected to respond within six weeks [2], and 60% within 12 months [3]. Importantly, clozapine has anti-aggressive effects [4] and its use can play a vital role in efforts to reduce the use of restrictive interventions such as restraint and seclusion [5].

**Fig. 1:**
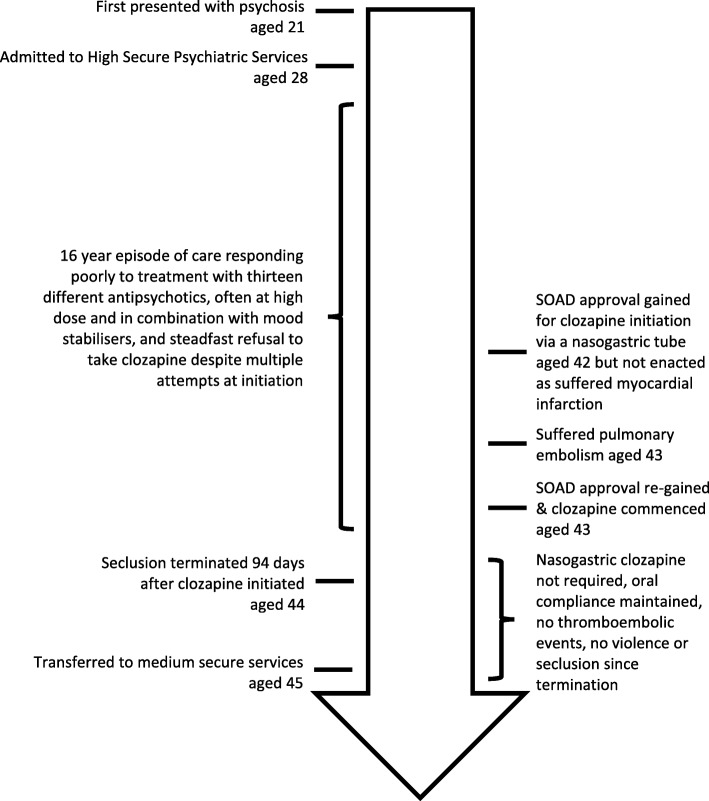
Timeline

Antipsychotics, and in particular clozapine, adversely affect the already poor cardiac and metabolic health of people with severe mental illness [6, 7]. There is increased risk of venous thromboembolism (at least 1 in 2–6000 [8]) and myocardial infarction [9]. Whilst thromboembolism is thought to be multifactorial in aetiology [10] and not restricted to clozapine alone [11], clozapine has the highest number of reported cases compared to alternative antipsychotics [12]. Despite these adverse effects, in people with schizophrenia, clozapine demonstrates the lowest all-cause mortality for those taking any or no antipsychotics [13].

Balancing the risks and benefits for non-compliant and non-capacitous patients is challenging, particularly at the extremes of what both society and the profession considers acceptable in terms of loss of dignity, autonomy and liberty, against imposing interventions which may cause life threatening adverse events.

## Case presentation

In this unique case, the impact of clozapine on a gentleman with treatment resistant schizophrenia, extreme violence requiring frequent physical restraint, and significant physical health concerns is described. Whilst there was a risk of significant and potentially life-threatening complications as a result of clozapine prescription, these were considered rare in comparison to the likelihood of improving the patients’ mental state and treatment was commenced.

The patient was a white British male. He first presented with psychosis at the age of 21. After entering the criminal justice system, he was transferred to high secure psychiatric services at the age of 28. He responded very poorly to treatment with thirteen different antipsychotics, including first and second generation antipsychotics both orally (including: chlorpromazine hydrochloride, flupentixol hydrochloride, thioridazine, haloperidol, droperidol, risperidone, olanzapine, amisulpride, quetiapine) and via intramuscular injections (including: pipotiazine palmitate, zuclopenthixol decanoate, haloperidol decanoate and fluphenazine decanoate). These were often at high dose and in combination with mood stabilisers (including carbamazepine, valproate, topiramate, lamotrigine). His compliance with oral medication was intermittent and he steadfastly refused to take clozapine despite multiple attempts at initiation. At most, he took clozapine for no more than one or two days.

Frequent episodes of psychotically driven violence towards staff, patients and his environment, including both assault with fists and a weapon, made his management highly problematic. He was managed in long-term segregation from 2010 onwards, and as a result of his behaviour his room required total refurbishment on more than occasion, each costing in excess of £10,000.

Alongside the physical health concerns related to his violence and the restraint necessary to control this and administer medication, he had numerous other physical health complications. He was noted to have: metabolic syndrome; a poor diet, a sedentary lifestyle, a Body Mass Index (BMI) of 31.5, poorly controlled lipids and had smoked heavily since childhood. However, despite these physical health concerns, an ‘assertive approach’ to clozapine was instigated [14]. This involved the potential administration of clozapine without his cooperation or consent via a nasogastric tube. At the time this patient was treated, an intramuscular clozapine preparation was not available in the UK. Approval by a Second Opinion Approved Doctor (SOAD), which is the legally sanctioned method in England and Wales [15], was gained.

During preparations to initiate clozapine, he suffered an inferior ST elevation myocardial infarction requiring two stents to his right coronary artery. He complied poorly with secondary prevention medications, accepting aspirin, but rejecting his second antiplatelet, beta-blocker, angiotensin-converting enzyme inhibitor and statin that had been prescribed by the cardiology team [16]. Complicating this treatment, he believed that the stents inserted were malign, repeatedly asking for them to be removed and felt that being in segregation meant being totally unable to participate in cardiac rehabilitation. Re-requesting SOAD approval following his myocardial infarction, clozapine was excluded from his subsequent treatment form whilst his cardiac rehabilitation and prognosis was being determined. Within the same year, he presented with increasing shortness of breath, and a computed tomography pulmonary angiogram (CTPA) scan confirmed that he had suffered a pulmonary embolism to the segmental branch of his pulmonary artery in his left upper lobe. His compliance remained poor. He continued to refuse medication, including warfarin, but ultimately accepted replacement low molecular weight heparin injections.

Non-compliance was suspected to be based on paranoid persecutory and somatic delusions. He believed that he had suffered a heart attack earlier in his life, that at times his heart had been removed and frequently declined blood tests believing that if he was told of an abnormality, then the worry would kill him. His mental state remained very difficult to treat. Additionally, he continued to complain of episodes of chest pain, although these were not thought to be cardiac in nature and subsequent electrocardiograms showed no further ischaemic events. A cardiologist was consulted about the cardiac risks involved. It was advised that after 6 months the stents would have epithelislised and therefore, the risks of a further cardiac event would have markedly reduced. As such, in collaboration, the cardiac and psychiatric team considered that the risks of both restraint and clozapine administration were not markedly increased comparatively to the baseline population rate and that alternative treatment approaches, including psychopharmacological and psychological therapies for his psychosis, were unlikely to succeed.

The team persisted with efforts to persuade the patient to comply with clozapine. With reassurance from cardiology, SOAD approval for nasogastric administration of clozapine was regained, but ultimately not required. He accepted oral clozapine within nine months of his myocardial infarction and a month from his pulmonary embolism, which was titrated as per British National Formulary guidance [17].

His mental state made a rapid and dramatic improvement. While his care was often traumatic, involving high levels of interpersonal conflict between the patient and his clinical team as we have described, he now maintains a good therapeutic relationship with them and is grateful for the care that he has received. After spending 1046 days in seclusion, this was terminated 94 days after clozapine initiation and has not been needed since. Both he and a relative were able to attend and actively contribute to an annual review, they were delighted with the treatment delivered. At the time of writing, he has been compliant with clozapine for 24 months, has had no incidents of violence during this time period, has complied with all physical health medications, and has been transferred to medium secure services, an overall timeline of his care is outlined in Fig. 1.

His physical health has stabilised despite continuing to lead a sedentary lifestyle and remaining obese (BMI 35). He developed hypertension and Type II Diabetes Mellitus shortly after clozapine was initiated. His triglycerides rose, reaching a peak of 22.2 mmol/L (normal healthy value < 1.7 mmol/L) in the same month after clozapine initiation: however, with pharmacological intervention, his plasma levels were recorded at 5.6 mmol/L a further 24 months later. He has had no further thromboembolic events. Chest pain continues intermittently but is well controlled with glyceryl trinitrate, and he has an extensive chest pain protocol in-situ which was jointly formulated with psychiatric and cardiology input.

## Discussion and conclusions

Although clozapine remains the only treatment likely to work in treatment resistant schizophrenia [18], psychiatrists remain anxious and at times reluctant to prescribe it, often on account of fears related to adverse drug effects and / or unfamiliarity with the drug [19].

This patient presented the team with an ethical dilemma [20].

Option A, of doing nothing (or more of the same) and accepting the status quo of a floridly psychotic man with a combination of severe physical and mental health problems, each seemingly interfering with the other, that would likely result in on-going segregation, further violence resulting in both risk to staff and to the patient during restraint, on-going refusal of the recommended drugs for management of his cardiac and pulmonary disorders, and unremitting psychosis.

Option B, of employing an assertive approach to the use of clozapine, which had a high chance of improving the patients psychosis and hopeful remission of his paranoid persecutory and somatic delusions which were limiting both his mental and physical health recovery, yet which also had uncertain but perceived lower risks related to restraint and drug effects.

This is a classic scenario for risk aversion [21], with further complications of the perceived difference in harms resulting from inaction when weighed against harms of actions deliberately chosen [22].

We posit that inaction in these circumstances and choosing to not deliver the most effective treatment, is fundamentally wrong. To deliver treatment successfully, a well functioning team is required, where trust and active cooperation between medical specialities (in this case both cardiology and haematology colleagues) and other clinical and non-clinical staff allows effective decisions to be made.

When psychiatrists ask for advice regarding physical health complications of psychiatric drug treatments, it can be tempting to try and outsource decision making. Yet while our colleagues in other medical specialities can give us advice on the risk assessment, monitoring and mitigation of physical health complications, it is us alone who can synthesise the risks and potential benefits, weighed against the practicalities of delivering treatment.

With this clinical picture and response to clozapine, its superior efficacy to alternative antipsychotics is clear and it has drastically improved the quality of life this patient leads. However, due to the potential for iatrogenic harm, clozapine remains underutilised [23], particularly where multiple physical health comorbidities are concerned.

When initiating clozapine, physical health and thromboembolic events must remain a significant concern for the clinical team but this must be carefully balanced against the patient’s mental state and risk factors intrinsically involved, both to themselves and others.

This case report is unique, as while it only highlights only one case of clozapine administration in the face of myocardial infarction, pulmonary embolism and hyperlipidaemia, it provides some reassurance that, despite significant concerns, clozapine remains a treatment option and can be safely prescribed with a significantly positive effect on both the psychiatric and holistic care of patients with treatment resistant schizophrenia.

## Abbreviations

BMI: Body Mass Index
SOAD: Second Opinion Approved Doctor
TRS: Treatment Resistant Schizophrenia
